# Type‐I‐interferon‐responsive microglia: participates in cerebral development and disease

**DOI:** 10.1002/mco2.629

**Published:** 2024-07-05

**Authors:** Hua Guo, Liyan Miao, Fangfang Zhou

**Affiliations:** ^1^ Institutes of Biology and Medical Science Soochow University Suzhou China; ^2^ Department of Clinical Pharmacology, The First Affiliated Hospital of Soochow University Suzhou China

1

A recent study conducted by Escoubas et al. published in *Cell* identified a population of type I interferon (IFN‐I)‐responsive microglia (IRMs) in the developing murine cortex.^1^ The study demonstrated a physiological role for IFN‐I‐driven whole neuronal microglial phagocytosis in brain development and function.

Microglia, specialized macrophages residing in the brain parenchyma, play a crucial role in the clearance of cellular debris, phagocytosis of invading pathogens, and modulation of neural circuit development. Microglia exhibit heterogeneity and spatial localization in both structure and function. Dysfunctions within distinct subsets of microglia are closely associated with the onset and progression of various diseases, yet the underlying mechanisms remain incompletely elucidated. Continuous advancements in sequencing technology and spatial omics have provided powerful tools for unraveling distinct microglial states. For example, A recent study employed single‐nucleus RNA sequencing and spatial transcriptomics to analyze the dorsolateral prefrontal cortex of female cynomolgus macaques exhibiting depression‐like behavior in response to social stress, revealing that changes in gene expression, mostly in microglia, are associated with depression‐like behavior.^2^

In order to further investigate the characteristics of microglia subtypes and their roles in neural circuits, the author performed single‐cell RNA sequencing on mouse cortical recovery phase cells. Interestingly, the sequencing results revealed a significant increase in the number of microglia expressing high levels of IFN‐I response genes. These findings suggest that IRMs play a crucial role in neural circuit remodeling. The authors simultaneously employed multiple independent clustering markers to validate interferon‐induced transmembrane protein 3 (IFITM3) as a specific marker for identifying IRMs.^1^ Subsequently, Escoubas et al. utilized IFITM3 to demonstrate the conservation of IFN‐I responsive microglia during cortical development, which transiently appear and persist in various pathological conditions such as neurodegenerative diseases, brain tumors, and viral infections. This provides a novel avenue for investigating the mechanistic involvement of microglia in various disease traits; however, further validation is required to ascertain the specific mode of action.

The cerebral cortex is a layer of gray matter on the outer surface of the brain and comprises six primary layers, denoted L1–L6, each exhibiting distinct cellular composition and functions. During the investigation of the form and positioning of IRMs, the author observed that IRMs' morphology differed from the typical ramified structure seen in the majority of cortical microglia. Specifically, the subtype located at L4 displayed an elongated shape and had the potential migration towards L5.^3^ L4/ L5 primarily consisted of excitatory neurons and a small population of inhibitory neurons that play a vital role in receiving sensory information from the external environment and transmitting it to other cortical regions.

During cortical development, microglia play a crucial role in sculpting neural circuits by engulfing and eliminating excessive or damaged neurons through phagocytosis. Escoubas et al. conducted a study to investigate the impact of IFN‐I signaling on this process. They observed that during cortical remodeling, IFN‐I‐responsive microglia were capable of engulfing neuronal somata by forming phagocytic cups around them. However, the authors' study was unable to determine which specific neurons were targeted for elimination, or whether they were phagocytosed while alive or after death. Subsequently, the authors examined the effect of loss of IFN‐I signaling specifically in microglia or neurons on the accumulation of neurons exhibiting DNA damage. Their findings confirmed that IFN‐I autonomously stimulates microglia to enhance their phagocytic function, suggesting that IFN‐I plays an intrinsic role in promoting microglial phagocytosis. To strengthen their investigation, the authors administered injections of IFN‐β into mice and Zebrafish models in vitro. The correlation between static biochemical measurements and dynamic fluorescence observations indicated that IFN‐I plays a critical role in promoting microglial phagocytosis and preventing the accumulation of DNA‐damaged neurons.

The IFN‐I response is essential for the initial antiviral defense of the body, exerting its effects through diverse mechanisms to inhibit viral replication and transmission, safeguard against viral infection, and enhance the antiviral capacity of host cells. Production of IFN‐I relies on activation of nucleic acid sensors such as mitochondrial antiviral signaling protein (MAVS) for recognition of cytoplasmic dsRNA, cyclic GMP‐AMP synthase (cGAS) for detection of double‐stranded DNA (dsDNA), and Toll‐like receptor 3. In Parkinson's disease, pathogenic progression was facilitated by α‐synuclein aggregates inducing DNA damage response in microglia and activating the cGAS‐STING pathway, resulting in an innate IFN‐I response.^4^ However, the author demonstrated that MAVS plays a crucial role in the expansion process of IRMs rather than cGAS. Loss of MAVS leads to significant accumulation of DNA‐damaged neurons. A limitation of Escoubas et al.’s study is their failure to analyze the mechanisms underlying dsDNA sensing, which presents a new avenue for future research.

Finally, the author conducted a whisker nuisance assay to gain further insights into the role of IFN‐I signaling in microglia. The results showed that both microglial‐specific deletion and whole‐body deletion of the Ifnar1 gene in mice led to a significant increase in tactile sensitivity and avoidance behavior when exposed to probes. Specifically, mice lacking IFN‐I receptor 1 (Ifnar1^‐/‐^) exhibited markedly heightened withdrawal responses to light touch on their whiskers, demonstrating heightened tactile sensitivity (Figure 1).

**FIGURE 1:**
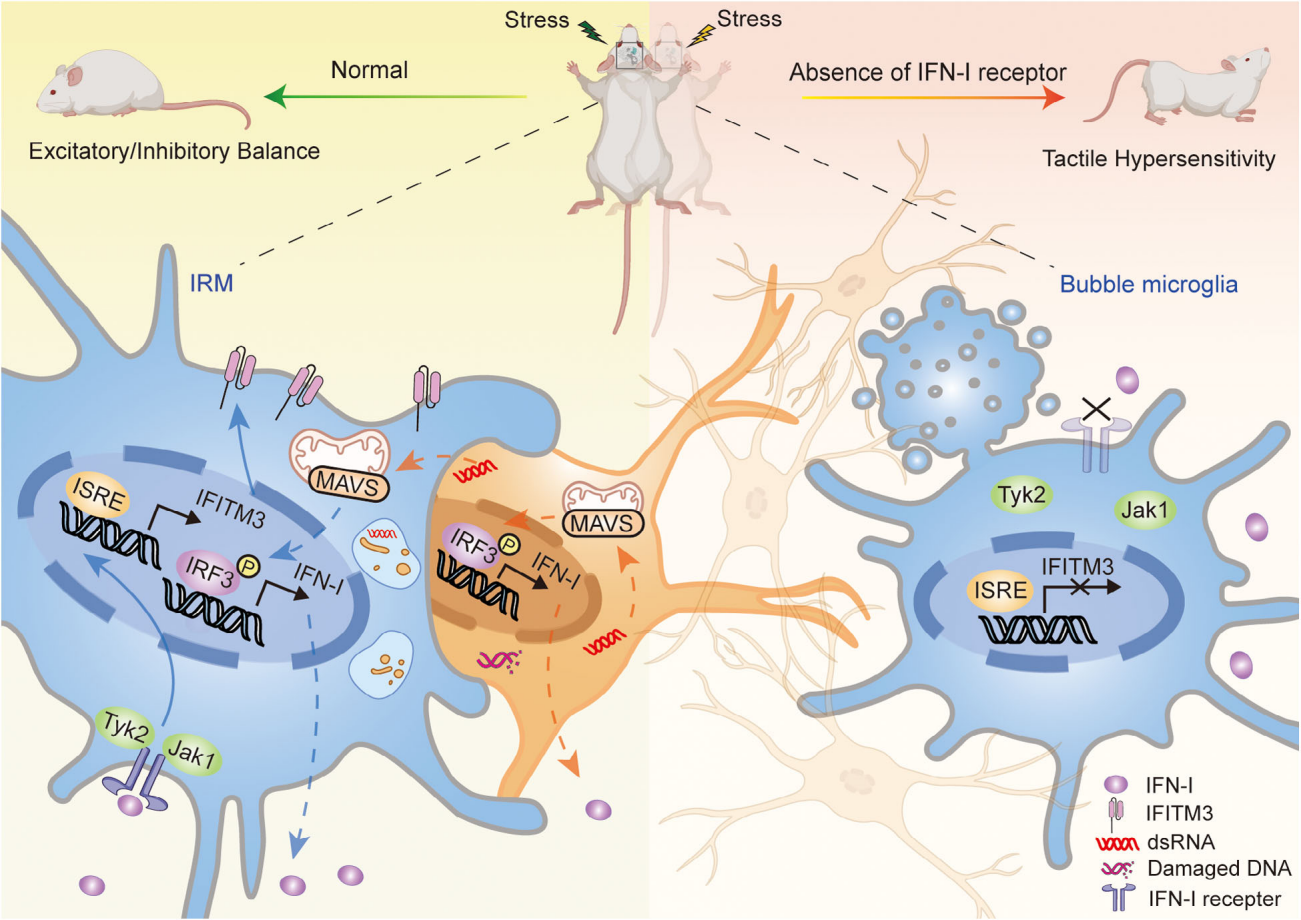
Type‐I‐interferon‐responsive microglia depend on IFN‐I to phagocytose DNA‐damaged neurons, thereby affecting cortical development and behavior. During early brain development, Type‐I‐interferon‐responsive microglia expand in response to external stress, which is enriched in type‐I‐interferon (IFN‐I) response genes, such as interferon‐induced transmembrane protein 3 (IFITM3). These microglia contain prominent IFITM3 phagocytic cups to phagocytose DNA‐damaged neurons in order to maintain excitatory and inhibitory neuronal balance. Global or microglial‐specific loss of the IFN‐I receptor resulted in microglia with phagolysosomal dysfunction and an accumulation of neurons with nuclear DNA damage, which are referred to as “bubble microglia”. Because double‐stranded DNA (dsDNA) breaks in neurons can also be observed with neuronal hyperexcitability, they often cause tactile hypersensitivity.

Traditionally, the IFN‐I response plays a pivotal role in the host's initial defense against viruses by inhibiting viral replication and transmission through multiple mechanisms. This protective mechanism shields the host from viral infection while enhancing the antiviral capabilities of host cells. However, in neurodegenerative diseases and other disorders, microglia also exhibit an indication of responsiveness to IFN‐I signals. Prolonged activation of IFN‐I‐responsive microglia may induce a hypersensitive phenotype, which significantly contributes to the progression of neurodegenerative diseases. For instance, Alzheimer's disease (AD) predominantly affects elderly individuals who often possess small populations of inflammatory and interferon‐responsive microglia within their aged brains.^5^ The high prevalence of early‐onset AD among individuals with Down syndrome can be attributed to the presence of both subunits (IFNAR1 and IFNAR2) encoding the IFN‐I receptor genes. Similarly, maternal immune activation has been epidemiologically linked to neurodevelopmental disorders.

In summary, based on previous research and the findings of Escoubas et al., IRMs represent a dynamic, intricate, and crucial subtype of microglial cell state. They play a pivotal role in bridging brain‐related neurodegenerative diseases, aberrant changes in IFN‐I levels, and brain development. Further investigation into IRMs may unveil additional molecular connections between immune system stimulation during development and neuropsychiatric disorders. These findings possess extensive applicability and significant implications for the exploration of novel targets or therapies aimed at treating and preventing neuropsychiatric disorders.

## AUTHOR CONTRIBUTIONS

H.G. wrote the manuscript and prepared the figure; F.Z. and L.Z. provided valuable discussion. All authors have read and approved the article. All authors have read and approved the article.

## CONFLICT OF INTEREST STATEMENT

The authors declare no conflict of interest.

## ETHICS STATEMENT

Not applicable.

## Data Availability

Not applicable.
